# Enzyme-Less Growth in *Chara* and Terrestrial Plants

**DOI:** 10.3389/fpls.2016.00866

**Published:** 2016-06-21

**Authors:** John S. Boyer

**Affiliations:** Division of Plant Sciences, College of Agriculture, Food and Natural Resources, University of Missouri, ColumbiaMO, USA

**Keywords:** calcium pectate, cell enlargement, *Chara corallina*, homogalacturonan, pectin, polygalacturonic acid, turgor, wall deposition

## Abstract

Enzyme-less chemistry appears to control the growth rate of the green alga *Chara corallina*. The chemistry occurs in the wall where a calcium pectate cycle determines both the rate of wall enlargement and the rate of pectate deposition into the wall. The process is the first to indicate that a wall polymer can control how a plant cell enlarges after exocytosis releases the polymer to the wall. This raises the question of whether other species use a similar mechanism. *Chara* is one of the closest relatives of the progenitors of terrestrial plants and during the course of evolution, new wall features evolved while pectate remained one of the most conserved components. In addition, charophytes contain auxin which affects *Chara* in ways resembling its action in terrestrial plants. Therefore, this review considers whether more recently acquired wall features require different mechanisms to explain cell expansion.

## Introduction

*Chara corallina* is a favorable model for studying how plants enlarge. Their cells contain no xylem, and phloem is absent in this particular species. There are no secondary walls characteristic of terrestrial plants, so lignin chemistry is at a minimum. Nevertheless, the walls contain cellulose, hemicellulose, and pectins like the primary walls of vascular plants (see discussion below). This review considers whether the growth mechanism in *Chara* might also be present in terrestrial plants.

Calcium complexing with pectin will be a prominent feature of the discussion and we will follow the practice of referring to Ca^2+^ when it is clearly ionized but spelling out calcium when it is complexed. This is because the complexes involve chelation whose coordination bonds do not conform to the valence state of the ion. The exact nature of the coordination bonds is debated and appears to involve stereochemical interactions with oxygen in the pectin that replace water in hydration shells around the Ca^2+^, typically estimated to be about eightfold bonding (Braccini and Perez, 2001).

The central feature of the growth mechanism is that *C. corallina* appears to enlarge its cell wall without the mediation of enzymes and instead in a strictly chemical fashion. This is based on three kinds of evidence. First, irreversible turgor-driven enlargement occurs in isolated walls (no cytoplasm). Second, the enlargement is observed after boiling the isolated walls for 10 min (no enzyme activity). Third, an enzyme-less chemistry can be demonstrated to produce the same enlargement in the isolated walls as in the living cells (chemical mechanism). As part of this process, new wall material is deposited and allows the strength of the wall to be maintained while it enlarges. All of these tests were made without removing the cells from the medium in which they were grown. Importantly, although enzymes produce wall constituents and deliver them to the wall, the enzyme-less portion begins after delivery by exocytosis.

The focus is on the wall because it is the tough outer covering controlling the rate at which plant cells enlarge. The enlargement in turn controls most plant size and thus plant growth. The wall chemistry in *Chara* was treated in an earlier review (Boyer, 2009), but experiments with terrestrial species cannot use the same methods as with *Chara* for various reasons. Despite this drawback, certain features of growth are apparent both in the alga and in various terrestrial species, and these will be compared.

Because the charophytes are the closest extant algal relatives of the progenitors of land plants, *Chara* walls accordingly lack a few constituents thought to have evolved later (and described below, but see Popper and Tuohy, 2010; Popper et al., 2011; Leliaert et al., 2012; O’Rourke et al., 2015 for details). The new constituents add new dimensions to the process and make it necessary to consider whether growth occurs by newly evolved and intrinsically different mechanisms.

## Background

The enlargement of multicellular plants relies mostly on the rapid and irreversible enlargement of cells localized in or derived from meristems. After cells divide, water moves down a gradient in water potential and into each cell, expanding the tough, elastic cell wall irreversibly (Boyer and Silk, 2004). The wall first becomes soft enough to yield to turgor and prevent turgor pressure from becoming as high as it otherwise would. This creates a growth-induced water potential low enough to bring water into the enlarging cells, while turgor remains high enough to continue extending the walls irreversibly (Boyer, 1985). The entering water fills the newly extended volume.

But much of this hydraulic complexity can be avoided if individual cells are surrounded with water. The cells then have abundant water instead of having to rely on hydraulic transport through intervening small cells. In an early attempt at a study of this kind, Green et al. (1971) explored the relationship between turgor pressure and enlargement in *Nitella* internodes. *Nitella* is a charophyte alga with single-cell internodes large enough to measure pressure and growth simultaneously. Despite having to use primitive methods, this remarkable work suggested there might be factors that kept growth fairly constant when turgor changes were small. The control of this system and whether it was enzymatic was not further explored.

Eventually, Proseus and Boyer (2006b) showed that it was possible to isolate the internode walls of a similar large-celled alga *C. corallina* without removing the cell from the culture medium. Artificial turgor pressures were developed by injecting oil into the wall lumen vacated by the cytoplasm. Pressures were comparable to those in the living cell. The oil did not leak through the wall because it was held by the same hydrophobic forces that hold the plasma membrane in place. Any substance could be injected into the lumen at any pressure.

As long as turgor was at normal levels for the intact cell (about 0.5 MPa or 5 bar), the isolated walls grew at a normal rate for 1 or 2 h whether they had been boiled or not (Proseus and Boyer, 2006b). During this time, growth was irreversible and rapid, just like that in the intact cell when attached to the plant. After 1 or 2 h, most growth stopped while it continued in the live cells. Although growth seemed to be enzyme-less, something was missing from the isolated wall. There are many ingredients produced in the cytoplasm that could be missing from isolated walls. What specific molecules allowed growth for 1 or 2 h, then were depleted or missing when walls were isolated from the cytoplasm?

## Wall Structure

In order to answer this question, it is first necessary to consider the structure of the wall. The internode cells of *Chara* and *Nitella* surround a large coenocytic cytoplasm and the walls appear to be dense gels about 5 μm thick in which cellulose microfibrils are embedded. Growing walls have a longitudinal elastic modulus of about 70 MPa that increases to 200 MPa as the cells mature, indicating the wall is tough and strong (Proseus et al., 1999). Elongation occurs evenly over the entire internode.

According to Morrison et al. (1993) and O’Rourke et al. (2015), the walls contain 45–50% pectin, 15–25% hemicellulose, and 30–35% cellulose, which is similar to the composition of the primary walls in dicotyledonous land plants. The pectins have sugar compositions suggesting homogalacturonans (polygalacturonic acid or PGA), i.e., linear unbranched polymers of α-1,4-D-galacturonic acid sometimes with a small amount of rhamnose (usually 1–2%; Ralet et al., 2001; Fraeye et al., 2009, 2010). The pectins also include rhamnogalacturonans (RGI) but not the boron-containing rhamnogalacturonan II (RGII) of terrestrial species (Matsunaga et al., 2004). The carboxyl groups of the PGA show little esterification and are mostly free to deprotonate to form a carboxylate anion (Anderson and King, 1961a,b; Morrison et al., 1993). The lack of esterification was detected by chemical means.

Recent genomic analysis indicates that genes for pectin synthesis diversified as plants acquired the land habit (Yin et al., 2010; Yang et al., 2013; McCarthy et al., 2014). For example, only a few homogalacturonan biosynthesis genes (α-galacturonosyltranserases (GAUTs) or GAUT-like) were detected in green algae but 10 were present in the moss *Physcomitrella patens*, 26 in sorghum (*Sorghum bicolor*), and 55 in soybean (*Glycine max*; Yin et al., 2010). Many genes related to pectin modification appeared to have diverged before genes for cellulose biosynthesis (McCarthy et al., 2014). Recently evolved walls of some grasses differ in pectin composition from those of dicotyledonous species mostly by containing less pectin and instead substantial amounts of glucuronylarabinoxylans and mixed linkage glucans (β1,3- and β1,4-linked glucose; (Vogel, 2008). It should be noted that glucuronylarabinoxylans might have pectin-like properties that could complement the low pectin contents of these grass walls. Levesque-Tremblay et al. (2015) review pectin modifications and point out some of the tissue-specific roles for them.

Among matrix polysaccharides other than pectins, *Chara* internode walls contain galacturonans, glucans, mannans, and xylans but none of the xyloglucans prevalent in terrestrial species (Popper and Fry, 2003), although xyloglucans were detected in the antheridia (Domozych et al., 2009). It seems likely that some of the matrix polymers have xyloglucan-like properties. In addition to these matrix polysaccharides, the cellulose in *Chara* is laid down in microfibrils mostly perpendicular to the long axis of the cell. The cells tend to elongate cylindrically (Baskin, 2001, 2005). These basic features are similar to those in land plants and because wall evolution in charophytes appears to have occurred monophyletically (Popper et al., 2011), growing *Chara* walls are likely to be primitive versions of their terrestrial counterparts.

## Basic Chemistry of Calcium Pectate

As described more fully below, the growth ingredient missing from isolated walls of *Chara* appeared to be newly secreted pectate. It combines with calcium that is the main inorganic constituent of the wall although smaller amounts of magnesium are also present (B, Na, K, Fe, Zn, and Cu could not be detected; Proseus and Boyer, 2006c). It is important to note that calcium forms pectate gels but magnesium does not.

This gelling activity is the basis for industrial applications of calcium pectate. Industrial quantities of PGA are obtained from citrus peel or apple pomace usually in an esterified form. Fraeye et al. (2009, 2010) point out that PGA from these sources contains methyl esterified galacturonic acid or occasionally in sugar beet or potato tuber acetyl esterified galacturonic acid. If the pectin is highly methoxylated, acid conditions cause a gel to form in the presence of a high concentration of sucrose. These conditions reduce electrostatic repulsion between polymers and decrease the water activity to form weak gels of jams and jellies. If the pectin is less methoxylated and Ca^2+^ is present, stronger gels form with calcium cross-bridges between pectin polymers.

Fraeye et al. (2009, 2010) also indicate that PGA can be demethoxylated either by chemical methods or enzymatically with various pectin methylesterases. The pectin methylesterases from terrestrial plants create clusters of galacturonic acid in blocks along the polymer backbone. Those blocks with 6–14 galacturonic acid units have an “egg-box” structure with calcium held in each box, cross-bridging anti-parallel PGA chains with sufficient strength to form the junction zones of a gel. Greater demethoxylation creates a stronger gel. When the demethoxylation is kept constant, gel strength increases with PGA concentration and/or Ca^2+^ concentration.

Basically, PGA acts as a chelator to hold divalent cations with coordination bonds. In order to form cross-bridges with calcium, the carboxyl groups need to be dissociated, and galacturonic acid in PGA has a pKa of about 3.5 indicating a weak acid. The acid weakens further as the degree of dissociation of the carboxyl groups increases (Ralet et al., 2001). Greater methyloxylation tends to return the pKa toward 3.5.

The pH has a moderate effect. Above 4.5, the gel properties are relatively independent of pH. Below 4.5, dissociation decreases and the affinity for Ca^2+^ decreases.

## Basic Chemistry of Pectates in Charophytes

Like charophytes, the primary cell walls of angiosperms include cellulose microfibrils synthesized by rosettes in the plasma membrane (Delmer and Amor, 1995; Sarkar et al., 2009; Gu et al., 2010). The microfibrils strengthen the composite wall structure but have crystalline diameters of only around 2–4 nm (Davies and Harris, 2003; Sarkar et al., 2009). These diameters are smaller than the water-filled interstices of the matrix embedding the microfibrils. The interstices are typically 3.5–8.6 nm in a range of species (Read and Bacic, 1996) including *Chara* (4.6–4.7 nm, Proseus and Boyer, 2005). It seems that without hemicelluloses, the microfibrils would be unable to remain in place. In effect, hydrogen bonding of xyloglucans (or their equivalent) to the microfibrils converts them to macrofibrils and anchors them in the matrix.

In contrast to cellulose biosynthesis, matrix polysaccharides (pectins and hemicelluloses) are released to the wall in vesicles matured from Golgi bodies (Zhang and Staehelin, 1992). The exocytosis is not influenced by turgor pressure because the release creates a thin “periplasm” or “cushion” separating the vesicles from the load-bearing part of the wall (Proseus and Boyer, 2006a). Pressures would be uniform across the vesicles as they release their contents to the wall. At normal turgor pressures in *Chara* (0.5 MPa), the periplasm is quite thin and the polymers are concentrated against the inner wall face (Proseus and Boyer, 2005). At these concentrations, pectins spontaneously form weak gels and if Ca^2+^ is present, make strong gels. At lower pressures, the periplasm is thicker because its osmotic properties cause water to be absorbed and thus dilute the periplasm.

The pressure causes the carbohydrates to enter wall interstices depending on their molecular weight. Dextrans (α-1,6-glucans) applied to the inside of the wall and mimicking the cytoplasmic delivery of matrix polysaccharides such as PGA (α-1,4-galacturonans) did not move into the wall unless driven by a pressure differential (Proseus and Boyer, 2005). The polysaccharide end (having the diameter of a sugar) appeared to start and the rest of the polymer reptated through in snake-like fashion. Small polymers (40 kDa) entered more readily than large ones (500 kDa), placing the small ones in intimate contact with polymers of the existing wall. This allows the polymers to bind to the existing wall and probably accounts for its general assembly.

Domozych et al. (2014) explored wall assembly in the charophyte *Penium margaritaceum* that constructs its wall in layers starting midway along the length of the cell. They report that a cellulose layer is deposited first and that pectin (homogalacturonan and RGI) is extruded through the layer onto the surface where it binds to Ca^2+^ to form a reticulated outer layer. They suggest that a similar process might account for the position of the middle lamella, with large carbohydrates percolating through the cellulose layer. The middle lamella is the primary cement holding plant cells together in a tissue. Perhaps like *Chara*, a turgor-dependence and reptation exists for pectin extrusion in *P. margaritaceum* and might be detected by varying the osmoticum around the cells (assuming osmotic adjustment is negligible).

The strength of the calcium cross-bridging and gelling activity varies with the number of cross-bridges. In the *Penium* system, calcium binding to pectin was enhanced by pectin methylesterase that increased the availability of carboxyl groups. The pectin lattice in the outer layer was more clearly reticulated at higher Ca^2+^ concentrations, indicating that calcium increasingly bound to the pectins in the layer. In the *Chara* culture medium containing 0.6 mM Ca^2+^, the walls had a moderate number of cross-bridges, allowing the walls to elongate. Increasing the Ca^2+^ to 50 mM decreased the rate of growth slightly (Proseus and Boyer, 2006c). If new pectate was then supplied to the culture medium containing 50 mM Ca^2+^, it combined with the calcium and immediately gelled, encasing the wall. The gel was so strong that it prevented further growth (Proseus and Boyer, 2006c).

The strength of these coordination bonds in cell walls is fertile ground for further work. So far, it appears that gel strength depends on the number of bonds in each local site and is somewhere between the strength of covalent bonds and van der Waals forces, i.e., in the range where the activation energy of enzymatic reactions also occurs in biology. This suggests that enzyme-mediated growth would be difficult to distinguish from enzyme-less growth unless stringent tests are made as described above.

## Unique Cyclic Chemistry Occurs in the Wall

**FIGURE 1:**
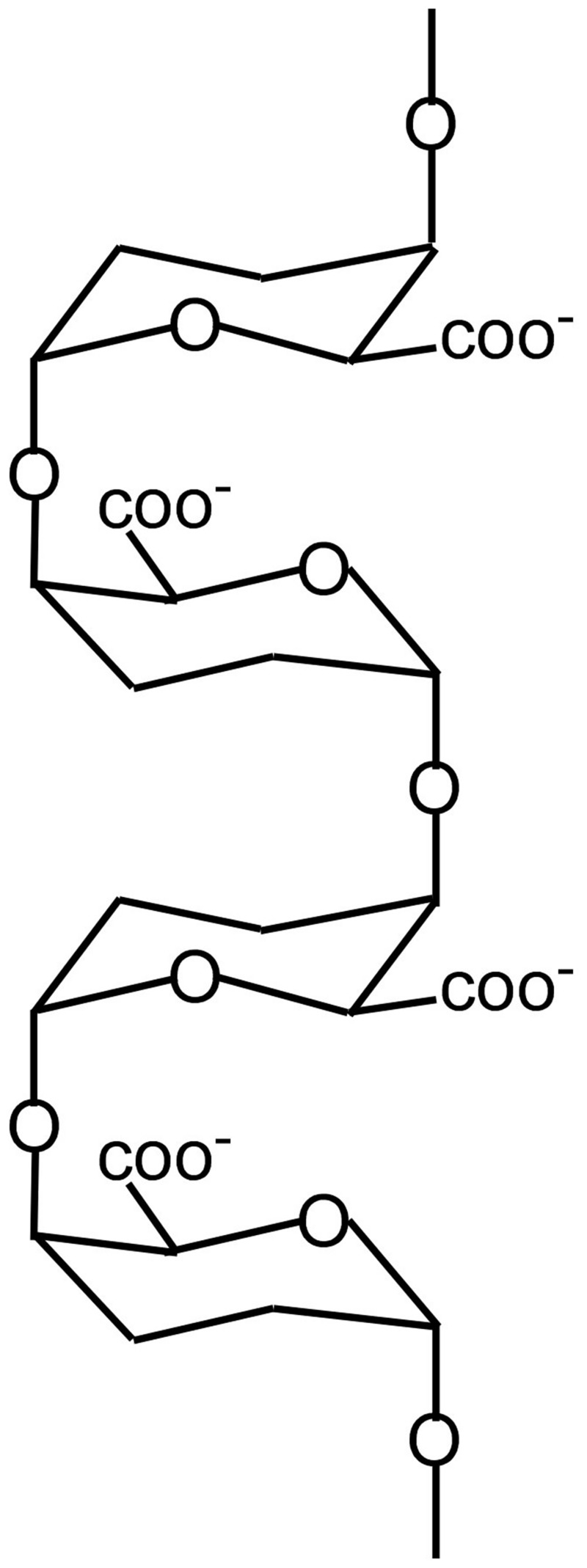
**Structure of PGA showing two egg boxes.** For simplicity, only oxygenic bonds and carboxyls are indicated.

**Figure 1** shows the basic structure of PGA (Ridley et al., 2001) and it is obvious that tension on the ends of the polymer will move the carboxyls apart, changing the strength of their coordination bonds with calcium. Morikawa and Senda (1974) and Morikawa et al. (1974) measured infrared spectra in *Nitella* cell walls under tension and found that the tension caused changes in the spectra of carboxyl groups. Similarly, turgor pressure puts wall polymers under tension in the plane of the walls, potentially distorting some of them. Clearly, those tensile elements bearing most of the load will be the most severely distorted and therefore the most susceptible to new PGA that is not distorted. Thermodynamically, this suggests that the egg-box structure in calcium pectate in the wall will lose its calcium to undistorted PGA whose bonds with calcium may be stronger. In this way, the new PGA from exocytosis can target the load-bearing population of calcium pectate in the wall. There is evidence that less than 5% of the calcium pectate in the wall is load bearing, distorted, and thus a target for new PGA (Proseus and Boyer, 2006c).

Of course below the minimum turgor pressure for distortion, there is no preferential targeting of the calcium pectate in the wall by the newly released PGA. Although the new PGA still removes some calcium from the wall, it has little effect on the extension of the wall (shown by Proseus and Boyer, 2007). The new pectate has growth activity only if the turgor pressure is above a minimum, i.e., creates enough tension to distort load-bearing wall pectate and creates a thermodynamic difference in bond strength with calcium.

**FIGURE 2:**
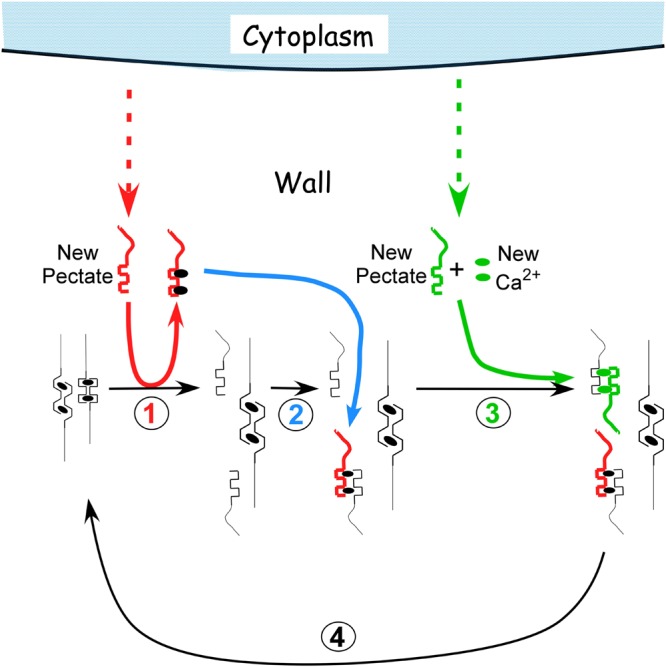
**Calcium pectate cycle.** Only two calcium pectate cross-links are shown (ovals in anti-parallel pectate molecules, left side of figure in black), but the same principles apply to larger numbers of calcium pectate cross-bridges in the wall. Turgor pressure is high enough to distort the egg box in one of the pair, weakening its bonds with calcium (left pectate in pair). New pectate from the cytoplasm (dashed red arrow) is undistorted and preferentially removes calcium from the distorted pectate (step 1, red). The load-bearing pectate relaxes after its cross-bridging calcium is removed. The wall elongates incrementally, shifting the load to the other member of the pectate pair, which distorts. The remaining steps 2–4 follow (see text) and result in a cycle. Additional calcium pectate is deposited in the wall as part of the cycle (step 3, green). The net result is elongation plus wall deposition. Note that in *Chara* the cycle occurs in the medium in which the cells are grown (0.6 mM Ca^2+^). Also note that the rate of growth depends on the rate of pectate release from cytoplasm to wall by exocytosis (red and green dashed arrows).

The distortion starts a sequence of reactions resulting in a cycle summarized in **Figure 2** (see Boyer, 2009 for more details). Starting on the left, turgor pressure above a threshold distorts the egg box for the load-bearing fraction of the pectate in the wall (**Figure 2**, left member of the pair). Exocytosis delivers new pectate to the wall. Because the new molecules are not under tension and are not distorted, they bind calcium more tightly than the distorted load-bearing pectate in the wall (**Figure 2**, step 1, red). This thermodynamic difference causes the new pectate to remove calcium from the distorted pectate preferentially. The vacated pectate in the wall relaxes and the wall elongates until other pectate takes the load and distorts (**Figure 2**, step 1, right member of the pair). The new pectate with its calcium can enter the wall and bind to the relaxed pectate (**Figure 2**, step 2, blue). This replaces the number of cross-bridges originally present but does not further strengthen the lengthened and thinner wall. Additional pectate from the cytoplasm plus Ca^2+^ from the medium is needed and binds to the rest of the vacated pectate (**Figure 2**, step 3, green). The deposition of this new calcium pectate re-establishes the original strength and thickness of the wall. However, this re-establishment creates the distorted pectate (step 1) and wall strength (step 3) to allow the reactions to repeat, completing the cycle (**Figure 2**, step 4, black). Each step in **Figure 2** has been verified experimentally (Boyer, 2009).

Tension is the key to this cycle sequence, and tests done without sufficient tension are unlikely to detect the sequence. But when the tension is above the minimum and cytoplasm releases new PGA to the wall, wall extension accelerates to the rate in intact plants. PGA of various molecular weights and from different sources has the same accelerating action. Pectic material is then deposited while the cell is growing (Proseus and Boyer, 2006a).

It is also important that cell enlargement without step 3 (**Figure 2**) would eventually result in a wall too weak to withstand turgor. Proseus and Boyer (2012a,b) tested this possibility and reported that the walls eventually burst when the cycle occurred without step 3. Bursting was abrupt and lethal for live cells.

The cycle shows that calcium chelation is dynamic, with bonds breaking and re-forming continually in the wall. In effect, the calcium pectate in the wall is in equilibrium with free Ca^2+^ in the interstices of the wall, and the calcium pectate gel continually re-structures whether new PGA is present or not. Above the minimum turgor pressure for growth but without new PGA, the wall may only “creep” slowly (distorted wall PGA gives up its calcium to a non-distorted wall PGA nearby). This auto-propagation probably accounts for any slow changes in length of isolated *Chara* walls after 1 or 2 h, i.e., in isolated walls no longer growing. Below the minimum turgor pressure for growth, auto-propagation is less likely and may not occur at all.

It is also worth mentioning that new PGA delivered by exocytosis is coming from a cytoplasm low in Ca^2+^ (concentration of less than 1 μM as a secondary signaling molecule). The newly released PGA is encountering the first substantial Ca^2+^ it has seen. Accordingly, Ca^2+^ migrates to the newly arrived PGA ultimately driving the elongation/deposition process. Because of the turgor pressure, PGA (now calcium pectate) supplied by the cytoplasm also can enter the wall and might cause growth in part by occupying space between the existing wall polymers, i.e., spreading the wall polymers apart by intussusception. This would cause wall pectin to distribute throughout the wall. Matrix polysaccharides distribute in this way in walls of rapidly growing tissues of terrestrial species (*Avena* and *Pisum*; Ray, 1967).

## Terrestrial Plants

One of the arguments favoring an enzyme-based growth process is the reaction to metabolic inhibitors, which decrease rates of expansion. However, many chemical inhibitors cause turgor loss in *Chara* (Proseus and Boyer, 2006b) and a better one may be low temperature, which inhibits growth with only a slight turgor loss due to its effect on the osmotic potential of the cell. At 0.1°C, *Chara* growth was zero but revived when turgor was raised above that normally in the cell (Proseus et al., 2000). It seems possible that the wall was capable of elongating but was not receiving new pectate from the cytoplasm because of the cold, as shown by Proseus and Boyer (2008). In this situation, the calcium pectate cycle would operate only slowly thus requiring higher turgor.

Tissues of most land plants do not have large cells as easily accessible to experimental manipulation as those of *Chara*. Furthermore, each cell may have its own turgor. Coleoptiles or intercalary meristems of hypocotyls tend to be more uniform and help minimize some of this complexity but, as mentioned above, the turgor must be high enough to expand the wall while still being low enough to bring water into the expanding cells. Turgor may thus increase or decrease depending on the treatment. Moreover, inappropriate turgor in only a few cells can change water potential gradients rapidly and thus availability of water to all the other cells, affecting growth (Passioura and Boyer, 2003; Boyer and Silk, 2004).

**FIGURE 3:**
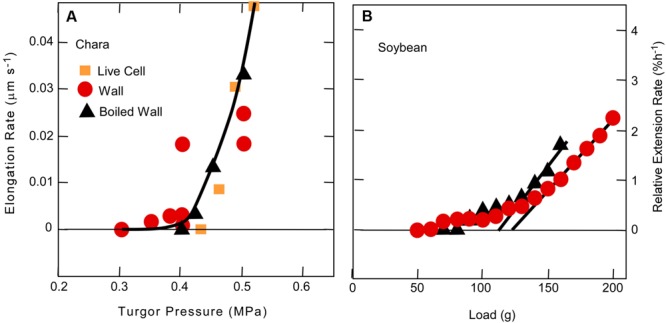
**Cell elongation in single *Chara corallina* internodes **(A)** or single soybean hypocotyls **(B)** when tension on the wall was varied.** In **(A)**, tension was varied with turgor pressure and in **(B)** with an extensometer. Data in **(A)** are for live cells, isolated walls, or isolated walls after boiling and in **(B)** for dead tissue or dead tissue after boiling. Although there was a slight difference in boiled and non-boiled tissue in **(B)**, it was not statistically significant. Note in both experiments that tension had to be above a minimum before irreversible extension was observed. **(A)** from Proseus and Boyer (2006b) and **(B)** from Ezaki et al. (2005).

In order to avoid this ambiguity, tissues may be killed. A unidirectional force is then applied in a direction the tissue would grow when alive, using an extensometer like that of Cosgrove (1989) that allows the extension to be monitored while the force is present. Above a minimum force, the tissue elongates irreversibly in proportion to the applied force just as in *Chara* internodes exposed to various turgor pressures (**Figure 3**). Ezaki et al. (2005) used this system to detect the minimum force for irreversible wall extension of soybean hypocotyls and found no statistical difference between dead tissue before or after boiling when the pH was 6 (**Figure 3B**). This result resembles that in *Chara*. But at pH 4–5.5, Ezaki et al. (2005) observed significant differences. Glutarate buffer was used for these latter experiments, and it is difficult to be certain but glutarate chelates calcium weakly and in the concentrations used (10 mM) at low pH, probably removed considerable calcium from the tissue. In this situation protons might more easily displace load-bearing calcium cross-bridges, accounting for the differences at low pH (see discussion of calcium pectate chemistry above).

In the same study, Ezaki et al. (2005) removed wall calcium with the chelator Quin 2 and observed an increased rate of irreversible extension. Addition of Ca^2+^ quenched the chelator activity. They could not observe the effect unless tension was above a threshold. Although Ezaki et al. (2005) did not expose the killed tissues to PGA or explore the effects in live tissues, they concluded that calcium cross-bridging of wall pectins accounted for their results. Essentially their results agree with the calcium pectate cycle of **Figure 2** except that Ezaki et al. (2005) also include a possible involvement of enzymes.

## Role of Xyloglucans, Proteins, and Enzymes

Xyloglucans have been proposed to bind cellulose microfibrils by hydrogen bonding, thereby controlling the expansion of cells (Passioura and Fry, 1992; Passioura, 1994). In effect, the xyloglucans were considered to be the main load-bearing wall components and the protein expansin was considered to release the xyloglucan bonds bearing the load (McQueen-Mason et al., 1992; McQueen-Mason and Cosgrove, 1995).

Zhao et al. (2008) found that cucumber hypocotyls lost their sensitivity to expansins as the hypocotyls matured. Zhao et al. (2008) were able to restore it with various hydrolytic enzymes targeting pectins (pectin lyase, pectate lyase, and polygalacturonase). Other enzymes had little effect (endoglucanases, xylanases, and protease). The sensitivity could be restored by treatment with the calcium chelator EGTA to remove wall calcium. They found more calcium in basal tissues than elongating tissues and conclude that calcium cross-bridging of pectates caused the loss of expansin activity in the basal tissues.

In *Arabidopsis*, Dick-Perez et al. (2011) used advanced NMR methods to study cell wall components and reported that xyloglucans seem less important as tethers of cellulose than are the pectins. They used double and triple mutants lacking xyloglucans that were developed by Cavalier et al. (2008) and Zabotina et al. (2012). The mutant plants showed morphological changes and grew a little less rapidly than the wild-type plants (Xiao et al., 2016). However, it is important that they did grow – and with no detectable xyloglucan. The authors concluded that other matrix polysaccharides may control the growth of the cells. When wall properties were examined in these mutants, the results were incompatible with a load-bearing xyloglucan (Park and Cosgrove, 2012a,b). Although Passioura and Fry (1992) and Passioura (1994) had suggested that load-bearing xyloglucan-cellulose tethers might control growth rate, the tethers might be replaced by calcium pectate cross-bridging with the same effect. Peaucelle et al. (2012) recognized this possibility and included calcium pectate in their model of wall expansion, suggesting it might act as in **Figure 2**. In this regard, it is of interest that *Chara* internodes grow without benefit of xyloglucans (Popper and Fry, 2003) or expansins (Cosgrove, 2000; Popper et al., 2011). Expansins are thought to have evolved after plants colonized the land (Popper et al., 2011).

According to **Figure 2** for *Chara*, growth rate is controlled by the secretion rate for PGA. Evidence for this concept also was found in *Arabidopsis* mutants impaired in secretion of PGA that displayed stunted growth (Mouille et al., 2007). Because the remaining PGA was methyl esterified, the authors propose that PGA synthesis was linked to methoxylation of the PGA, and the mutants disrupted this part of the synthesis and thus exocytosis. Gendre et al. (2013) found mutants inhibiting Golgi secretion of matrix polysaccharides and observed severe dwarfism in shoots and roots. Moreover, Zhu et al. (2015) found major losses in pectin deposition that led to decreased growth. Caffall et al. (2009) report not only decreased growth but also lethal effects of some pectin mutants of *Arabidopsis*. In another test, Kong et al. (2015) modified xyloglucans in an *Arabidopsis* mutant by deleting galactose. They observed inhibited rates of secretion resulting in slower growth, i.e., dwarfism. With an additional mutation to eliminate xyloglucans altogether, vesicle secretion reverted nearly to the wild type and so did growth rate. The authors concluded that altered xyloglucan was more inhibitory to secretion than elimination of the xyloglucan. But the experiment was also a test for the rate of secretion of matrix polysaccharides. It thus appears that the exocytosis of matrix polysaccharides is a central feature of the growth rate of this plant.

Instead of xyloglucans, Derbyshire et al. (2007) focused directly on pectins and recognized that decreasing methyl esterification of PGA might increase the calcium cross-bridging in the walls, strengthening them and decreasing their extensibility. They expressed a fungal pectin methylesterase in *Arabidopsis* to lower the degree of methyl esterification and reported slower growth presumably from the stiffened walls. In the reverse experiment, pectin methylesterase mutants of *Arabidopsis* unable to remove the methyl esters caused the walls to weaken (Hongo et al., 2012). Also, maintaining high levels of methylesterification in transgenic *Arabidopsis* increased the size of the plants as expected if the walls were highly extensible, i.e., had few calcium pectate cross-links (Lionetti et al., 2010). Interestingly, Xiao et al. (2014) found polygalacturonase in the apoplast of *Arabidopsis* and conclude that it aids cell expansion in hypocotyls when over-expressed. Each of these papers suggest the fundamental control of cell enlargement involved the pectins.

## Auxin

In general, growth regulators affect cell enlargement but most are beyond the scope of this review. However, in evolutionary terms, auxin is an ancient regulator of plant development (Cooke et al., 2002) and it mediates many of the processes in charophytes also seen in terrestrial species. In *Chara*, internodes etiolate in low light. Growing plants also show apical dominance, develop rhizoids more rapidly if auxin is present (Klambt et al., 1992), and display rapid basipetal transport of the hormone (Boot et al., 2012; Zabka et al., 2016). Charophytes appear to control auxin concentrations by a balance of biosynthesis and degradation rather than by conjugation with other molecules as in many terrestrial species (Cooke et al., 2002). Some but not all of the auxin signaling pathways have been detected in algae, suggesting that additional auxin functions may have evolved as plants moved onto land (De Smet et al., 2011).

## Acid Growth

Auxin accelerates cell elongation and is most obvious in certain susceptible tissues such as coleoptiles and hypocotyls. A prominent theory is that proton ATPases in the plasma membrane are stimulated by auxin, creating a low pH at the inner wall face and causing “acid growth”. The increased acidity was proposed to enhance the growth rate of the tissue, and low pH did so transiently (Rayle and Cleland, 1972, 1992). The concept was easily tested in *Chara* because the culture medium had enough intrinsic buffering capacity to hold pH nearly constant while cell elongation was measured without additional buffers. Efforts to demonstrate an acid effect failed (pH as low as 3.5, Proseus and Boyer, 2006c).

Virk and Cleland (1988, 1990) studied growth in soybean hypocotyls and concluded that there were acid labile, load-bearing bonds in the wall, but Ezaki et al. (2005) questioned whether applied tensions were high enough to cause irreversible wall extension. Nevertheless, it is worth noting that methoxylated pectate is less acidic than pectate without methoxy groups (see above description of pectate chemistry). Peaucelle et al. (2008) report that localized de-methylesterification and thus acidification is required for organ formation in *Arabidopsis* meristems. By placing beads with pectin methylesterase on the meristem, floral primordia could be induced where none would ordinarily exist. Growth of the stems was not affected, so the local acidification did not extend to the whole meristem. In addition, Peaucelle et al. (2015) found that local acidification controlled growth anisotropy in *Arabidopsis* hypocotyls. The idea that de-methylesterification increases extensibility is the opposite of the usual interpretation that de-methylesterification increases calcium cross-bridging and decreases extensibility. Instead, Peaucelle et al. (2008) suggest more extensible walls might be caused by too little Ca^2+^ available locally.

Braybrook and Peaucelle (2013) have evidence that local auxin triggers local cell wall softening in meristems, initiating organ development. However, auxin is also required for general extension of the primordium. This implies that there is a transition from a local effect to a general one as the organ develops, oriented around cell wall properties. The concept that a patterned signal transitions to a general one deserves further attention.

## Peroxidase and Hydroxyl Radicals

A totally different view of growth control by auxin involves breakage of covalent bonds by reactive oxygen intermediates, specifically hydroxyl radicals (Schopfer, 2001; Schopfer et al., 2002; Liszkay et al., 2004). There is no doubt that these intermediates could break covalent bonds in the wall because of their high reactivity (Fry, 1998). An obstacle is the absence of oxidases necessary to form the intermediates, such as superoxide dismutase, oxalate oxidase, ascorbate peroxidase, glutaredoxin, and most peroxidases that are not present in root tips of maize (Zhu et al., 2007). On the other hand, reactive oxygen intermediates have been implicated in the formation of lignins (Passardi et al., 2004) and since maturing tissues of terrestrial species generally have some lignin-containing cells, the associated chemistry is likely to be present (Schopfer, 1994; Voothuluru and Sharp, 2013). Reactive oxygen might signal developmental changes that trigger lignification. Incidentally, *Chara* and *Nitella* do not develop secondary walls like those of terrestrial plants, and peroxidase chemistry is less prominent (Passardi et al., 2004). As land was invaded by plants, lignins and other forms of secondary wall thickening evolved depending on the species. This probably occurred because the colonization of land made gravitational force a larger feature than in the algae, and secondary thickening became necessary to strengthen the walls after the primary cell walls had expanded.

Tagawa and Bonner (1957) took a different view of the auxin mechanism of growth control and tested the effects of Ca^2+^, Mg^2+^, and K^+^ on auxin-accelerated elongation of *Avena* coleoptiles that contained mostly primary walls. The authors observed large effects of Ca^2+^ and were among the first to suggest pectins could be involved. In related work about the same time, Heath and Clark (1956) exposed wheat coleoptiles to chelators or auxin and found similar effects for both types of molecule. In fact, chelators sometimes were considered to have hormonal properties (Heath and Clark, 1956; Weinstein et al., 1956). Consistent with these papers, the accelerating action of 10 μM auxin was inhibited by Ca^2+^ in maize coleoptiles (Siemieniuk and Karcz, 2015). No chelators were used and instead the authors measured membrane polarization which changed with auxin and Ca^2+^ and K^+^ treatment. They propose that the changes in polarization were attributable to H^+^-ATPase activity in the plasma membrane, linking coleoptile growth to auxin action by acid growth. Although the polarization was correlated with auxin effects, no causation could be assigned and instead the authors indicated that Ca^2+^ might have altered K^+^-channel activity sufficient to change the polarization and growth rate. However, it seems possible that an alternate explanation could be the pectin one of Tagawa and Bonner (1957). Further work with this coleoptile system would be valuable.

**FIGURE 4:**
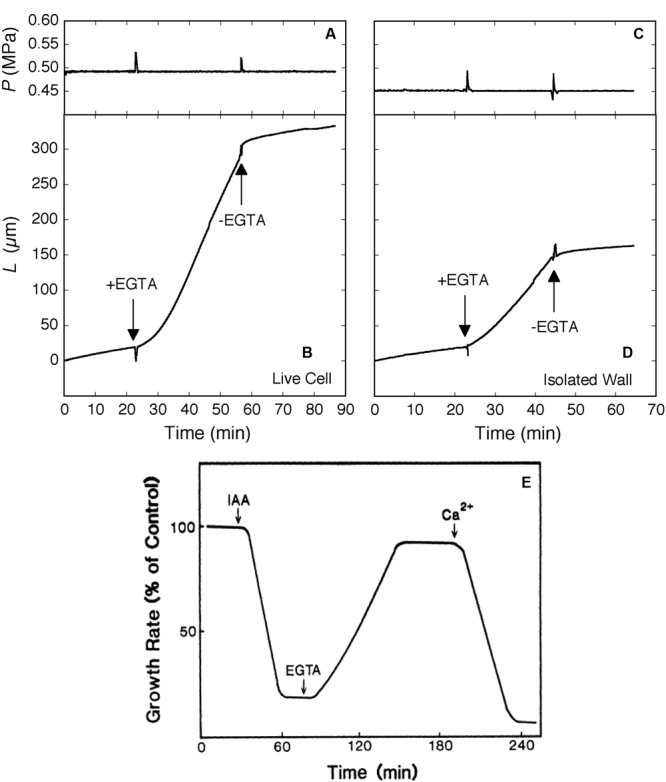
**Elongation in *Chara corallina* internodes **(A–D)** and maize roots **(E)** exposed to the Ca^2+^ chelator EGTA (downward arrows).** In *Chara*, the EGTA (2.5 mM, pH 7) removed 95% of Ca and 100% of Mg from the wall. Ca^2+^ (0.6 mM) was resupplied when EGTA was removed with fresh culture medium (upward arrows). Turgor pressure (*P*) is shown in **(A)** and **(C)**, change in length of internodes (*L*) in **(B)** and **(D)** for live *Chara* cells and isolated walls, respectively. In maize **(E)**, roots were exposed to a growth-inhibiting concentration of IAA (10 μM) and subsequently to EGTA (1 mM), then to Ca^2+^ (0.5 mM). Root growth is shown as % of control rate. **(A–D)** from Proseus and Boyer (2006c) and **(E)** from Hasenstein and Evans (1986) by permission from www.plantphysiol.org, Copyright American Society of Plant Biologists.

The sensitivity to auxin differs between shoots and roots. In maize, an auxin concentration of 10 μM that accelerated the growth of coleoptiles (Siemieniuk and Karcz, 2015) inhibited the growth of roots (Hasenstein and Evans, 1986). When the auxin-inhibited roots were exposed to EGTA, the inhibitory action of auxin was reversed (**Figure 4**). Ca^2+^ quenched the EGTA effect, restoring the auxin-induced inhibition. The authors interpreted these effects in terms of a secondary messenger role of calcium in the cytoplasm but the similarity to enzyme-less growth in **Figure 2** is striking.

**FIGURE 5:**
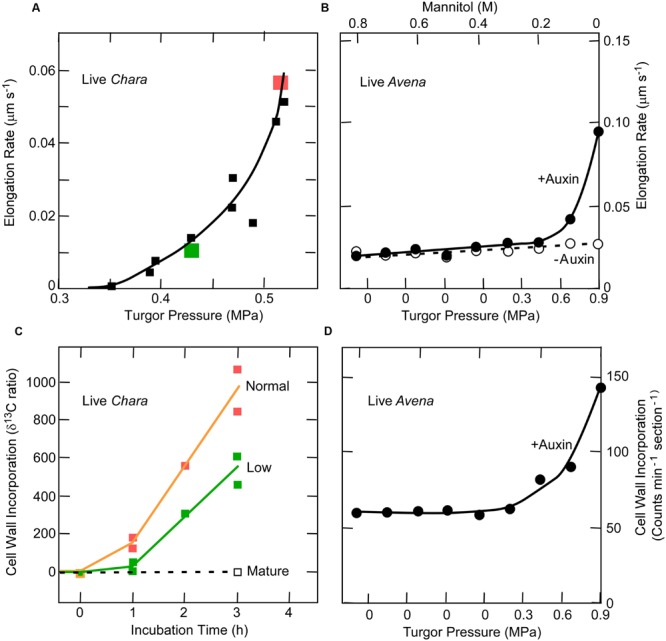
**Elongation and cell wall deposition at various turgor pressures in live *Chara* internodes and *Avena* coleoptiles. (A)** Elongation in *Chara*. **(B)** Cell wall deposition in *Chara* at normal and low turgor pressures. The normal turgor of about 0.5 MPa was decreased by 0.1 MPa using the pressure probe, as shown in red and green. **(C)** Elongation in *Avena* with and without 30 μM auxin at various turgor pressures. **(D)** Corresponding wall deposition in *Avena* with the 30 μM auxin. Data from Proseus and Boyer (2006a) and Cleland (1967).

Auxin accelerates both the elongation of coleoptiles and the delivery of pectin and other matrix polysaccharides to cell walls (Albersheim and Bonner, 1959; Baker and Ray, 1965; Ray and Baker, 1965). Consequently, it would be predicted that, at turgor pressures low enough to inhibit auxin-induced elongation, the deposition of new wall material would also be inhibited. This is indeed found in *Chara* as shown in **Figures 5A,B** (Proseus and Boyer, 2006a), but *Avena* coleoptiles also display a similar turgor dependence for the two processes (Cleland, 1967) (**Figures 5C,D**). This turgor dependence suggests a similar link might exist between wall deposition and growth in *Chara* and *Avena*.

The existence of this link is one of the stronger tests of the calcium pectate cycle. That the exocytosis of matrix polysaccharides, including pectins, was affected by auxin suggests a key role for the cycle in auxin-stimulated tissues. However, no reports are known to this author that include auxin-treated tissue exposed to PGA. The calcium chelator EGTA is perhaps the closest analogy (Hasenstein and Evans, 1986). The molecule is small enough to enter the interstices of the wall in most tissues while externally supplied PGA may be excluded because of its size (e.g., 170 kDa sometimes used by Proseus and Boyer, 2006c). When PGA is externally supplied, it is exposed only to atmospheric pressure instead of the higher pressure needed for molecular entry into the wall (Proseus and Boyer, 2005). But in the *Chara* experiments, even though externally supplied PGA might not enter the wall, the immediacy of its action suggests that wall calcium was removed instead. The Ca^2+^ ion would readily move through the wall to the PGA outside. External PGA of 170 kDa removed about 60% of the wall calcium in *Chara* (Proseus and Boyer, 2006c). Similar removal might be expected in terrestrial tissues exposed to PGA.

## Tip Growth

In contrast to *Chara* and many tissues of terrestrial plants, pollen tubes and root hairs grow at the tip rather than along the entire cell. In fact, pollen tubes grow more rapidly and for greater distances than any other cell. An example is maize, whose pollen tube travels about 10 mm per hour for about 300–400 mm. Calcium is necessary for pollen tube growth and below 10 μM, the cell bursts. Above 10 mM, the cell stops growing. Between these extremes, growth is rapid but may not be continuous because in lily the rate oscillates with a period of 15–50 s (see review by Hepler et al., 2013). The oscillation has been used to determine which events precede or follow the maximum rate.

The wall of the pollen tube is rich in pectins and in order to deliver the pectate to the tip, Golgi-derived vesicles containing methoxylated pectate are carried along actin cables that appear to oscillate in their delivery rate, possibly under the control of a tip-focused gradient in cytoplasmic Ca^2+^ where sub-μM concentrations act as a second messenger (Hepler et al., 2013). The vesicles fuse with the plasma membrane and release their contents to the wall by exocytosis. If maximum growth rates are used as a reference condition, the highly methoxylated pectate is released a few seconds before maximum growth occurs. This may be because the esterification blocks many of the carboxyls and only a few cross-bridges form when exposed to Ca^2+^. This causes a highly extensible tip and accounts for the maximum rate (McKenna et al., 2009).

**FIGURE 6:**
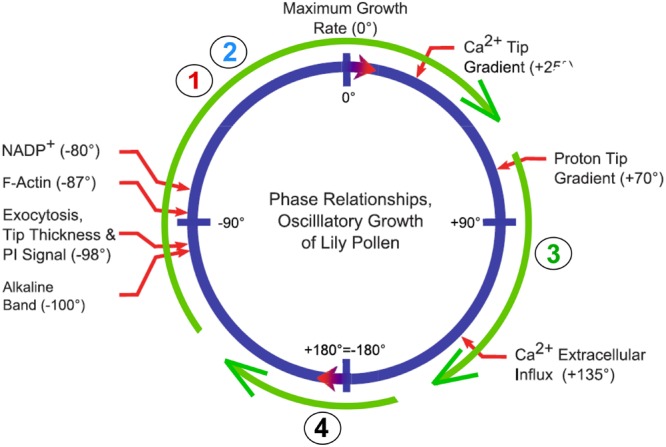
**Possible relation of calcium pectate cycle to oscillatory growth of a pollen tube.** Blue circle shows phase angles and thus sequence of events for pollen tube growth of lily from Figure 3 of Hepler et al. (2013). Green circle shows Steps 1–4 of calcium pectate cycle from **Figure 2**.

On the other hand, maximum Ca^2+^ uptake occurs a few seconds after the maximum growth (Hepler et al., 2013). At the tip, pectin methylesterases are also secreted and fail to be active for a few seconds. They are secreted as a pro-enzyme, and proteases in the wall act on the pro-enzyme to create the active form. Also, an inhibitor at the tip is retrieved by endocytosis behind the tip. Both of these actions delay the esterase activity until after the peak growth rate. As a result, most newly freed carboxyl groups are in the shank of the pollen tube. They bind calcium and stiffen the wall, as measured by micro-indentation (Parre and Geitmann, 2005; for alternate view, see Vogler et al., 2012). **Figure 6** shows how this oscillating action might relate to the *Chara*-type calcium pectate cycle of **Figure 2**. Rojas et al. (2011) also propose that a similar cycle controls pollen tube growth. They tested a physical model using rheological principles and conclude that the fit with calcium pectate chemistry is quite good.

## Other Forms of Targeted Wall Delivery

Another example of targeted wall delivery is seen in transfer cells. These cells develop specialized wall ingrowths that expand the area of the plasma membrane and allow more transporters to be present in the expanded area, enhancing transport across the membrane. The cells are particularly obvious between parent plants and their embryos, which require rapid transport as the seed develops. The ingrowths form from wall constituents delivered to the inner surface of specific areas of the primary wall. McCurdy et al. (2008) report that the ingrowths consist mostly of cellulose and matrix polysaccharides that resemble those already present in the primary wall. Their development seems similar to the mechanism of tip growth in pollen tubes, but with targets at specific areas of the inner face instead of being localized at a growing tip. Recent work indicates that Ca^2+^ plumes can be seen extending from the cell exterior into the cytoplasm at each targeted locus (Zhang et al., 2015a,b). It seems possible that the plumes expose PGA to Ca^2+^ before the PGA extracts calcium from the wall. Consequently the new pectate might be deposited immediately rather than act to remove wall calcium. This suggests that only step 3 of **Figure 2** might operate, accounting for the unusual anatomy of the transfer cell walls.

## Conclusion

The primitive walls of *Chara* and *Nitella* allow certain features of cell expansion to stand out, particularly pectin chemistry. The properties of pectin also are important for terrestrial plants but the evidence is arguably less direct than in the charophytes. Nevertheless, there are hints that the calcium pectate cycle may occur in both kinds of plants. Cell expansion is linked to wall deposition in both, and the two processes require turgor pressure above a minimum, rely on the rate of exocytosis, respond to pectin methoxylation, are affected by Ca^2+^, and share certain auxin features. Although *Chara* internodes expand more slowly than the cells of terrestrial species, expansion continues longer (weeks), and the final cell length equals or exceeds that of many terrestrial cells. Wall synthesis must keep up and emphasizes the important of the linkage between expansion and deposition.

In *Chara*, a unique aspect of calcium pectate chemistry is the distortion by turgor that leads to a cycle of reactions. If turgor diminishes slightly, the cycle can cease and the ensuing reactions also cease even though the plants appear moderately turgid. When normal turgor returns, the cycle can resume. It is tempting to explore this behavior more fully in terrestrial plants because it may be a tool for controlling or testing the presence of the cycle.

The evidence so far, while still fragmentary, suggests that the process in *Chara*, being chemical, could be widespread in green plants. Wherever the chemicals are present and turgor can distort the pectate, the reactions should occur. Pectins particularly homogalacturonans seem to be among the most conserved wall components in green plants (Ridley et al., 2001; Popper and Fry, 2003; O’Rourke et al., 2015). With adequate water, the cells should be able to generate enough turgor to run the cycle. Fueling this speculation is the cross-bridging of the pectate as part of its chemistry. This structural feature immediately suggests a means to loosen or tighten a wall.

In turn, it seems that methoxylation may be a means to control the cross-bridging, usually acting as a growth accelerant when enhanced but a decelerant when diminished. The evidence is strong in pollen tubes but the opposite has been reported when organs are initiated in *Arabidopsis* meristems. This suggests that pectin has roles beyond the calcium pectate cycle, and recently evolved pectin modifications probably reflect this.

Methoxylation is minimal in *Chara* perhaps because the single large wall must have high strength to bear the tension of turgor pressure (pressure is force/area and the larger the area the larger the force). Plants with small cells are found among the charophytes and these show evidence of methoxylation (e.g., Domozych et al., 2014). In tissues of terrestrial species, small cells are the rule and many walls share the tension. Perhaps methoxylation evolved to control cross-bridging and thus the rate of growth in these plants.

Later evolutionary arrivals such as RGII complexed with boron may play a role in growth. Boron deficiency can be especially obvious in meristems of terrestrial species and a mutant for a B transporter causes deformed meristems that alter the development of reproductive and vegetative tissues in maize (Durbak et al., 2014). Although RGII is a pectin, no direct tests have been made of its possible growth effects. This leaves open the possibility that later-evolved features of wall pectins could participate in meristem function as well as wall biomechanics.

Because the calcium pectate cycle links elongation and wall deposition of pectins automatically without special regulatory metabolism, it would be useful to know whether the link is similar in terrestrial plants. If so, it may be unnecessary to invoke recently evolved but fundamentally different mechanisms for cell enlargement.

This link indicates that prolonged intervals of low turgor necessarily must signal metabolism. Otherwise, wall constituents would accumulate unused in the cytoplasm or wall. In *Chara*, the feedback requires 23–53 min after which biosynthesis is brought into balance with the lower rate of deposition of matrix polysaccharide (Proseus and Boyer, 2008). The signals controlling this process are intriguing and need further identification.

## Author Contributions

The author confirms being the sole contributor of this work and approved it for publication.

## Conflict of Interest Statement

The author declares that the research was conducted in the absence of any commercial or financial relationships that could be construed as a potential conflict of interest.
